# Structures of MPND Reveal the Molecular Recognition of Nucleosomes

**DOI:** 10.3390/ijms24043368

**Published:** 2023-02-08

**Authors:** Meiting Yang, Xiaorong Li, Zizi Tian, Lulu Ma, Jun Ma, Yunlong Liu, Guohui Shang, Ailing Liang, Wei Wu, Zhongzhou Chen

**Affiliations:** State Key Laboratory of Agrobiotechnology, College of Biological Sciences, China Agricultural University, Beijing 100193, China

**Keywords:** MPND, MPND–DNA complex, crystal structure, histone, nucleosome

## Abstract

Adenine N^6^ methylation in DNA (6mA) is a well-known epigenetic modification in bacteria, phages, and eukaryotes. Recent research has identified the Mpr1/Pad1 N-terminal (MPN) domain-containing protein (MPND) as a sensor protein that may recognize DNA 6mA modification in eukaryotes. However, the structural details of MPND and the molecular mechanism of their interaction remain unknown. Herein, we report the first crystal structures of the apo–MPND and MPND–DNA complex at resolutions of 2.06 Å and 2.47 Å, respectively. In solution, the assemblies of both apo–MPND and MPND–DNA are dynamic. In addition, MPND was found to possess the ability to bind directly to histones, no matter the N-terminal restriction enzyme-adenine methylase-associated domain or the C-terminal MPN domain. Moreover, the DNA and the two acidic regions of MPND synergistically enhance the interaction between MPND and histones. Therefore, our findings provide the first structural information regarding the MPND–DNA complex and also provide evidence of MPND–nucleosome interactions, thereby laying the foundation for further studies on gene control and transcriptional regulation.

## 1. Introduction

The DNA N^6^-methyladenine (6mA) modification is a well-known epigenetic mark in prokaryotes. It is also understood to help discriminate between cellular “self” DNA and invasive “non-self” DNA [1,2,3]. Recently, with the development of detection technologies, 6mA has also been detected in eukaryotes [4,5,6,7], although its biological functions in eukaryotic cells remain to be further elucidated. By identifying domain homologs of prokaryotic origin, the restriction enzyme-adenine methylase-associated (RAMA) domain was predicted as a candidate 6mA sensor in eukaryotes [8]. Moreover, the Mpr1/Pad1 N-terminal (MPN) domain-containing protein (MPND) was the first reported protein containing the RAMA domain to directly bind to DNA with the 6mA modification in vitro [9]. Nevertheless, the precise identification mechanism remains to be discovered. In addition, the RAMA domains are frequently fused to the deubiquitinating peptidase (DUB) domains in eukaryotes, such as in Myb-Like, SWIRM, and MPN domains 1 (MYSM1) [10]. It is worth noting that MPN, another domain of MPND, is also responsible for DUB activity.

The MPN domain is widespread in archaea, bacteria, and eukaryotes. In eubacteria and archaea, the MPN domain is usually found in single proteins, whereas, in eukaryotes, the MPN domain is either part of a polypeptide chain or the subunit of several multiprotein complexes. Most of the studied MPN domain-containing proteins are linked to different signal pathways, such as DNA damage repair [11], transcriptional regulation [10], protein synthesis, and degradation [12,13]. However, the function of MPND, which is a member of the MPN super-family proteins, has yet to be discovered. Additionally, MPND has been found to be linked to gastric cancer; yet, it is currently unknown how MPND and carcinogenesis are related [14,15].

In this work, we investigated the first crystal structures of the apo–MPND and the MPND–DNA complex from *Mus musculus* at the atomic resolution. Next, we confirmed that the assembly of apo–MPND was dynamic, and that MPND molecules independently bound to DNA in solution. Most importantly, our study showed that histones interact directly with the two major structural domains of MPND (RAMA and MPN) in vitro. The DNA and two acidic regions of MPND synergistically enhance the interaction between MPND and histones. To summarize this point, our results provide the first structural insights into the MPND–DNA complex, as well as evidence for MPND–nucleosome interactions, thus helping to reveal a new mechanism of gene control.

## 2. Results

### 2.1. Crystal Structure Determination and Characterization of apo–MPND

**Figure 1:**
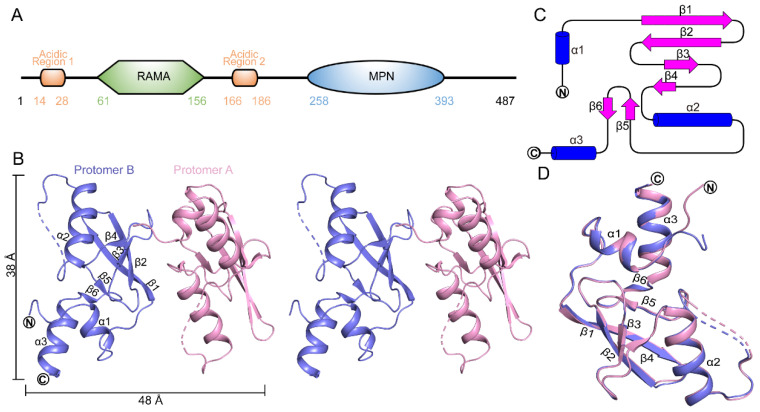
Crystal structure of the apo–MPND RAMA domain. (**A**) Domain architecture of the MPND. MPND comprises acidic region 1, RAMA domain, acidic region 2, and MPN domain. Residue numbers indicate domain boundaries. (**B**) Stereoview of the apo–MPND structure. The two MPND protomers are represented in slate and pink, respectively. (**C**) The topology of the MPND RAMA domain. In addition, the representation of α-helices, magenta; β-sheets, blue; and loops, black lines. (**D**) Superposition of two protomers in the apo–MPND structure.

We attempted to study the roles of MPND in binding DNA by expressing and purifying a series of truncated MPND fragments in *E. coli* (Figure 1A and Figure S1). After extensive crystallization trials and optimizations, we successfully obtained MPND (residues 2–160) crystals suitable for diffraction experiments (Figure S2). The structures were determined via the molecular replacement program BALBES [16] using the CcrM structure (PDB ID: 6PBD) as the model [17]. The final apo–MPND structure in the space group *P*2_1_2_1_2_1_ was refined to 2.06 Å resolution, with an *R_work_* of 23.0% and an *R_free_* of 24.9%. Data collection and refinement statistics are shown in Table 1.

In the final structural model, the asymmetric unit contained two protein protomers (Figure 1B), each with three helices (α1–α3), two long antiparallel β-strands (β1–β2), and four short β-strands (β3–β6) (Figure 1C). Each protomer included MPND residues 62–156 with missing residues (131–133). According to the SDS-PAGE of crystals, the key reason the N-terminal structure could not be resolved was due to the conformational flexibility during crystallization rather than due to protein degradation (Figure S3). The two protomers possessed virtually identical spatial arrangements, as revealed by structural superposition (Figure 1D), with an overall root-mean-square deviation (RMSD) of 1.0 Å for all Cα atoms. The main structural differences between the two protein protomers were in the irregular loops, particularly in the N-terminal location. A DALI search [18] of the apo–MPND structure identified the RAMA domain of the DNA methyltransferase protein (CcrM, PDB ID: 6PBD, Z = 10.4, and RMSD = 1.9 Å) as the closest structural homolog (Figure S4) [17]. Additionally, the sequence alignment from different proteins showed that most residues in the RAMA domain were not conserved (Figure S5). This, therefore, suggested that the structure of the RAMA domain was evolutionarily conserved and had little to do with the sequence.

### 2.2. Specific Recognition and Binding of dsDNA by MPND

**Figure 2:**
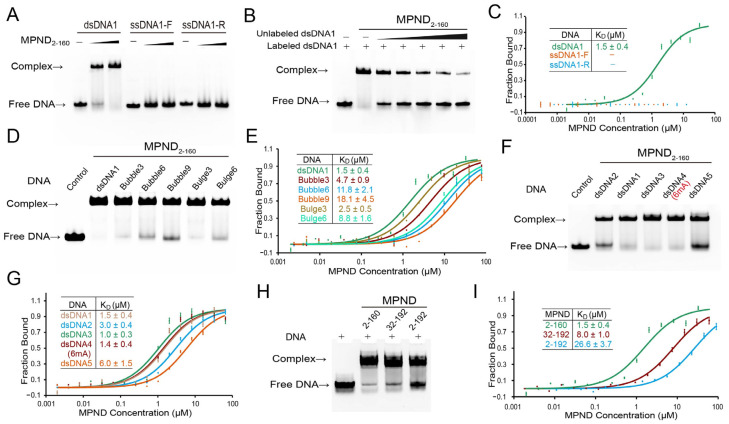
The binding of MPND to dsDNA. (**A**) The EMSA of MPND with dsDNA and ssDNA. Arrows indicate the positions of free DNA or protein-binding DNA. (**B**) EMSA showed that the binding of MPND with FAM-labeled dsDNA1 was reduced by the unlabeled DNA. The EMSA assay was detected by the fluorescence of FAM. (**C**) MST measurement of the binding affinity of MPND with DNA. (**D**) The MPND bound to general dsDNA or bubble/bulge DNA containing different amounts of mismatched bases in the middle of dsDNA. (**E**) MST determined the binding affinity of MPND to general dsDNA or the bubble/bulge DNA containing different numbers of mismatched bases in the middle of dsDNA. (**F**) The EMSA of MPND bound to dsDNA with different lengths or with 6mA modification. (**G**) The measurement of the binding affinity of MPND with dsDNA of different lengths or 6mA modification by MST. (**H**) The EMSA of different MPND truncates with the dsDNA. (**I**) The measurement of the binding affinity of MPND truncates with dsDNA by MST. These experiments were all repeated three times.

Earlier research suggested that the MPND binds to double-stranded DNA (dsDNA) containing 6mA modifications in vitro [9]. However, the exact mechanism is still unknown. We first carried out electrophoretic mobility shift assays (EMSA) in order to confirm the binding between MPND and DNA. According to the EMSA data, MPND bound directly to dsDNA, but not to ssDNA (Figure 2A). Furthermore, unlabeled dsDNA was utilized as a competitor to rule out the potential of non-specific binding and fictitious interactions. The signal of the FAM-labeled complex diminished as the competitor increased, thereby indicating that the binding between MPND and dsDNA was actual and specific (Figure 2B). Meanwhile, the equilibrium dissociation constants (*K*_D_) were determined by microscale thermophoresis (MST) experiments. In addition, the MST results consistently showed that MPND bound to dsDNA with a *K*_D_ of 1.5 ± 0.4 μM, and ssDNA did not exhibit a detectable binding signal (Figure 2C). The binding affinity assays for different bubble or bulge DNA further supported this conclusion. When compared with normal dsDNA, the binding affinity decreased gradually with the increased number of mismatched bases in the middle of dsDNA (Figure 2D,E). Then, various double-stranded oligonucleotides ranging in length from 15 to 42 bp were used to test the effect of DNA length on binding. Furthermore, the EMSA and MST experimental findings demonstrated that the length of the dsDNA fragment possessed no obvious influence on the binding (Figure 2F,G). In addition, we noticed that 6mA did not affect the binding of MPND to dsDNA.

A continuous length of acidic residues (14–28, 169–189) was observed both in the N-terminus and the C-terminus of the RAMA domain. For ease of use, the following sections will refer to these two regions as acidic region 1 and acidic region 2, respectively. Given that these two acidic regions are conserved in different species (Figure S1), we hypothesized that this arrangement would affect the DNA binding. Moreover, Figure 2H,I showed that both acidic regions inhibited the binding to DNA, but, surprisingly, the inhibition of acidic region 2 was markedly more potent than that of acidic region 1. This result was reasonable due to the fact that the acid residues in acidic region 2 were more concentrated and closer to the RAMA domain, leading to charge repulsion between the DNA phosphate backbone and the acidic regions. In addition, we performed the EMSA experiment with MPND by using different DNA sequences. The experimental results showed no substantial differences in MPND in terms of binding to different DNA sequences, thereby demonstrating that the DNA recognition by MPND might be sequence-independent (Figure S6).

### 2.3. The Complex Structure of MPND with Double-Stranded DNA

**Figure 3:**
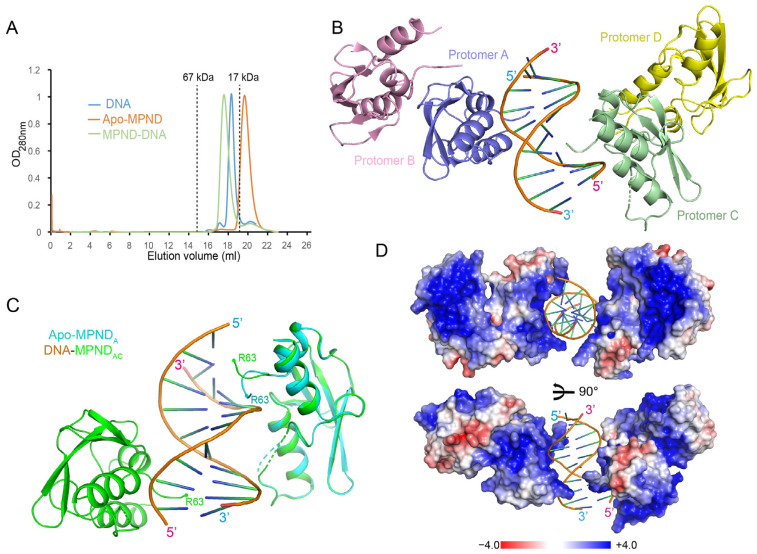
Structure of the MPND–DNA complex. (**A**) Size exclusion chromatography (SEC) of apo–MPND, DNA, and the complex on a Superdex^TM^ 200 Increase 10/300 GL column. Elution volumes of the protein standards are marked at the top of the figure. (**B**) The ribbon representation of the MPND–DNA complex structure. (**C**) The superposition of the apo–MPND (cyan) and the MPND–DNA complex (MPND, in green) structures. (**D**) The electrostatic surface potential of the MPND–DNA complex at ±4 kT/e: red (acidic), white (neutral), and blue (basic).

In order to elucidate how DNA is linked to MPND, we sought to determine the complex structure of MPND with a nucleic acid substrate. We used different MPND truncations with dsDNA to generate stable complexes for crystal screening and, fortunately, obtained the complex crystal for the fragment (residues 54–163) (Figure 3A and Figure S2). Then, the complex structure was solved using a molecular replacement procedure in CCP4 with the above-determined apo–MPND structure as a template [19]. In addition to the MPND structure, the primitive density map also included continuous electron densities that displayed a double-stranded nucleic acid profile (Figure S7). However, as the DNA recognition by MPND appeared to be sequence-independent (Figure S6), and the DNA bases were averaged in the structure determination, the final nucleic acid density could only correspond to 10 base pairs. Therefore, we modeled the adenine and thymine into the electron density. The complex was refined to 2.47 Å in the space group *P*1, with an *R_work_* of 24.5% and an *R_free_* of 25.4% (Table 1). The final refined complex structure contained four MPND molecules, as well as one dsDNA in the asymmetric unit (Figure 3B). The overall conformation of MPND between the apo- and DNA-bound forms was found to be similar, as demonstrated by the structural superposition, with the RMSD of all Cα atoms being just 0.8 Å (Figure 3C). Moreover, the electrostatic analysis showed that the protein surface in contact with DNA was positively charged, thus providing a favorable environment for DNA binding (Figure 3D).

### 2.4. Key Sites of Interaction between MPND and Nucleic Acid

**Figure 4:**
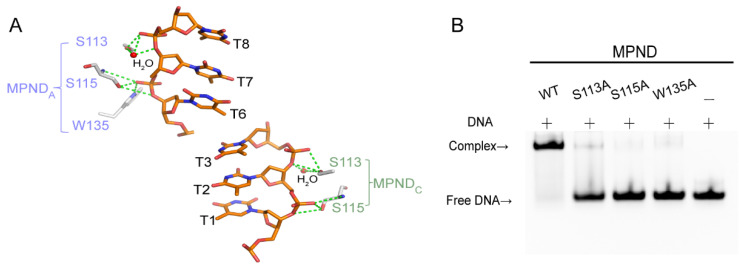
Interaction of MPND and dsDNA. (**A**) Close-up view of the interaction between key residues and DNA bases. (**B**) The EMSA of different MPND mutants with the DNA.

Our complex structure provided details of this interaction at the atomic level. Among the four MPND molecules solved in the asymmetric unit, only two MPND molecules were involved in direct contact with dsDNA. This binding was weak, with the total buried interface area being only 291 Å^2^ and 256 Å^2^, respectively. As shown in Figure 4A, the primary protein–DNA interactions between MPND and dsDNA were the hydrogen bonds formed between Ser113, Ser115, Trp135, and the DNA backbone. In addition, two water-mediated hydrogen bonds involving S113 were present there. However, no specific protein–base interaction was found. As such, we generated MPND point mutants to confirm the role of the above residues. In parallel, the EMSA experiments were used to determine the mutants’ qualitative differences with respect to DNA binding. All the mutants, namely S113A, S115A, and W135A, almost lost their DNA binding affinity when compared with the WT (Figure 4B).

Additionally, the key residues in the MPND–DNA complex structure possessed lower temperature factors (Figure S8). When compared with the apo–MPND structure, the orientation of the N-terminal loop changed upon binding to dsDNA, and R63 moved over the major groove of dsDNA (Figure 3C). In conclusion, these results imply that these critical residues contribute to dsDNA binding.

### 2.5. The Oligomeric State of apo–MPND and MPND–DNA Complexes in Solution

Two MPND molecules were present in the asymmetric unit of the apo–MPND structure, but their interaction was weak (Figure 1B). In order to investigate the oligomeric state of apo–MPND in solution, we analyzed the apo–MPND structure using the protein interfaces, surfaces, and assemblies (PISA) server [20]. Interestingly, the structural analysis suggested two possible dimer configurations: dimer 1, which possessed an interface area of 439 Å^2^; and dimer 2, which was generated from the symmetry and possessed an interface area of 411 Å^2^.

**Figure 5:**
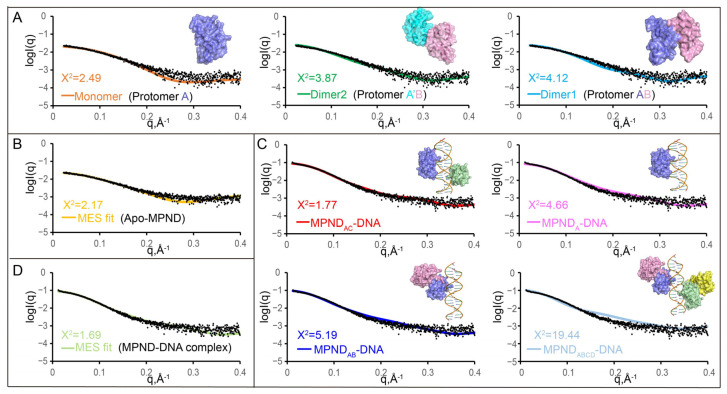
Analysis of the apo–MPND and MPND–DNA in solution via the SAXS (small-angle X-ray scattering) experiment. (**A**) Comparison of the SAXS experimental data (black dots) and the calculated scattering profiles for apo–MPND at 1.25 mg/mL. (**B**) Comparison of the SAXS experimental data (black dots) and the theoretical scattering curves of apo–MPND from the MES fit. (**C**) Comparison of the SAXS experimental data (black dots) and the calculated scattering profiles for the MPND–DNA complex at 1.25 mg/mL. (**D**) Comparison of the SAXS experimental data (black dots) and the theoretical scattering curves of the MPND–DNA complex from the MES fit.

Next, we used apo–MPND_54–163_ in a range of concentrations for small-angle X-ray scattering analysis (SAXS) to further study the oligomeric state of apo–MPND in solution. It must be noted that SAXS is an effective tool for examining dynamic components in solution, and it works well in conjunction with high-resolution techniques, such as X-ray crystallography. The monomer fitted the SAXS profile best at 1.25 mg/mL, followed by dimer 2, and dimer 1 fitted less well (Figure 5A). Meanwhile, a minimal ensemble search (MES) [21] was performed, as this could be very useful for analyzing mixtures in solution. A subset of conformation ensembles containing a monomer and dimers was selected to fit the experimental data. The ensemble of conformation mixtures containing both a dimer and a monomer fitted the data better than a single dimer or monomer (Figure 5B), revealing that the monomer was a major component in the solution. For other concentrations, the results were similar. Therefore, the oligomeric state of apo–MPND in the solution is mainly monomeric.

Then, we proceeded to examine the oligomeric state of the MPND–DNA complex in solution at different concentrations. The SAXS results suggested that the DNA complex containing two MPND molecules, rather than one or four, fitted best (Figure 5C). As the two MPND molecules in the MPND–DNA heterotrimeric complex (MPND_A_-dsDNA-MPND_C_) did not interact, it was implied that the MPND molecules might not have a cooperative effect. Consistently, the free energy of dissociation (ΔG^diss^) for the heterotrimeric complex (MPND_A_-dsDNA-MPND_C_) and the heterodimeric complex (MPND_A_-dsDNA) were 0.3 and 2.3 kcal mol^−1^, respectively, as calculated by the PISA server [20]. The small positive value of ΔG^diss^ indicates that the heterotrimer may be easily dissociated to one heterodimer and one MPND molecule. Similarly, MES was used to analyze the complex in solution and the MES fit was enhanced, thus indicating the presence of the heterodimeric complex in addition to the heterotrimeric complex (Figure 5D). When combining the results in the apo–MPND structure, both dimers and monomers are present in the solution, and, thus, may be dynamically changed in binding DNA.

### 2.6. Direct Histone Binding by MPND and Cooperative Regulation by Acidic Regions and DNA

**Figure 6:**
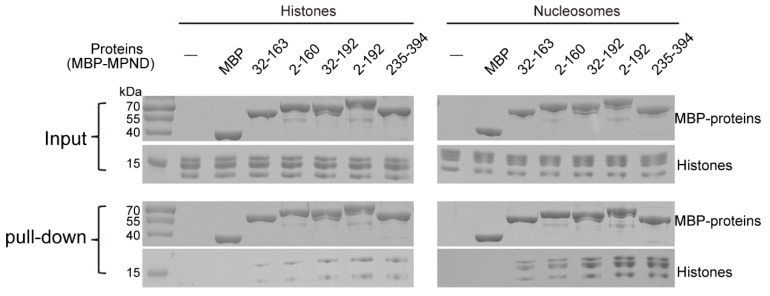
MPND interacts with histones or nucleosomes by MBP pull-down assay. The different domains/regions in the MPND fragments used are as follows. MPND_32–163_: RAMA domain; MPND_2–160_: acidic region 1 and RAMA domain; MPND_32–192_: RAMA domain and acidic region 2; MPND_2–192_: acidic region 1, RAMA domain, and acidic region 2; MPND_235–394_: MPN domain. The concentrations of MBP-MPND, histones, and nucleosomes used in the experiment were 0.5 μM, 2.1 μM, and 1.5 μM, respectively. Input and eluted proteins were analyzed by SDS-PAGE, followed by Coomassie Bright Blue staining. Note that MBP protein was taken as a control (CK).

We solved the structure of the MPND–DNA complex, and electrostatic analysis showed that the protein surface in contact with DNA was mainly distributed with positive charges. Interestingly, negative electrostatic was also found on the structure’s surface (Figure 3D). In addition, acidic regions 1 and 2 exist in front of and behind the RAMA domain of the MPND (Figure 1A), respectively. Due to the fact that the positive residues are rich in histone tails, we hypothesize that the MPND may also bind to histones directly. Furthermore, we conducted pull-down assays using the histones expressed in prokaryotic cells to verify our hypothesis. According to these pull-down assays (Figure 6, left panel), the standalone purified MPND RAMA domain (MPND_32–163_) interacted directly with the histones. Meanwhile, the two acidic regions promoted this binding as we predicted, with a more marked promotion by acidic region 2 (MPND_32–192_) than by acidic region 1 (MPND_2–160_). In agreement with this, MPND_2–192_ exhibited the strongest binding to histones. According to previous studies [10], the MPN domain-containing deubiquitinase (DUB) MYSM1 acted to remove monoubiquitin from Histone H2A (H2A-K119Ub). Given that MPND also contains the typical MPN domain, we speculate that the MPND’s MPN domain may also be recruited to histones. As shown in Figure 6, MPND_235–394_ also bound histones, thereby indicating that the lone MPN domain of MPND interacted with histones directly.

Prompted by these findings, we further examined the impact of dsDNA on MPND-binding histones. Notably, the binding of MPND with nucleosomes was stronger than that with histones alone (Figure 6, right panel). Nucleosomes increased the binding of MBP-tagged MPND to histones, with synergistic effects of two acidic regions, the RAMA domain and the MPN domain. In summary, MPND binds directly to histones. In addition, DNA further facilitates their binding, which provides a new strategy for studying gene activation and silencing pathways.

## 3. Discussion

Previously, there was very little information regarding the structure and function of MPND. Herein, we first clarified that MPND recognizes and binds dsDNA, not ssDNA (Figure 2A–C). Moreover, the mismatched bases in the middle of dsDNA reduce the binding. This substrate recognition by MPND might be sequence-independent (Figure S6). Due to the charge repulsion between the DNA phosphate group and the acidic residues, the two acidic regions inhibit the binding of MPND to DNA (Figure 2H,I). Then, the apo–MPND and MPND–DNA structures solved in this study first provided details of this interaction at the atomic level (Figure 1B and Figure 3B). For example, R63 is over the major groove of dsDNA (Figure 3C). In addition, the structural analysis revealed that S113, S115, and W135 were crucial for the MPND–DNA interactions (Figure 4A), which was further supported by mutation studies (Figure 4B). Through the SAXS experiments, we knew that the assembly of apo–MPND is dynamic in solution and mainly monomerized. Further, the binding of MPND molecules to DNA is independent (Figure 5). Consistently, in the crystal structure of the MPND–DNA complex, two kinds of MPND exist. Two MPND molecules bind dsDNA, while another two MPND molecules do not bind dsDNA and stabilize the crystal packing. Thus, MPND_D_ in the complex has conformational changes, such as the change in the α2 helix length (Figure S1). Consistently, the interface areas between these two MPND protomers were increased in the MPND–DNA complex when compared with those in the apo–MPND structure.

To date, CcrM is the only published structure including the RAMA domain. Despite the low sequence conservation between the RAMA proteins (Figure S5), the superposition of the structures of CcrM and MPND (Figure S4) implies that this RAMA domain structural core is conserved and provides an important reference for other unresolved proteins that contain the RAMA domain. Indeed, in the CcrM-DNA structure, the RAMA domain contacts DNA backbones without specific protein–base interaction [17]. This is consistent with what we observed in our structure, thus highlighting that DNA substrate recognition via the RAMA domain may be sequence-independent. Due to this, nucleic acids were averaged, resulting in the final complex structure’s lack of nucleic acid base density and, thus, a lower interface area. A previous study demonstrated that the MPND RAMA domain may bind selectively to dsDNA containing 6mA in a native sequence through the DNA pull-down assay [9]. However, in our work, we observed that the 6mA modification did not significantly increase MPND binding to DNA (Figure 2F,G). We speculate that MPND may collaborate with other proteins/nucleic acids to identify 6mA, which deserves further investigation.

Finally, we determined the direct interactions of MPND with histones. Moreover, the two acidic regions promoted the binding of MPND to histones. However, only H3/H4 were detected (Figure 6, left panel). Interestingly, the DNA in the nucleosomes further promotes the binding of H2A/H2B (Figure 6, right panel). Moreover, the MPN domain of MPND can also bind to H2A/H2B with the help of DNA. Additionally, DNA and the two acidic regions synergistically promote the binding of H2A/H2B. In this regard, Zhu et al. evaluated MPN domain-containing MYSM1 as a direct histone H2A deubiquitinase, thus revealing a new regulation strategy for gene activation [10]. Our results revealed that MPND possesses multiple functions, such as binding DNA and interacting with histones. These findings suggest that MPND may be related to histone ubiquitination, thus indicating a previously unrecognized form of gene control and transcriptional regulation.

Together, our results provide the first structural insights into the MPND–DNA complex and evidence of the direct interactions between MPND and histones. Most significantly, the dynamic assembly and unique features of MPND open up new avenues for research into epigenetics, gene control, and transcriptional regulation.

## 4. Materials and Methods

### 4.1. Cloning, Expression, and Purification of Protein

The restriction sites *Bam*H I and *Xho* I were used to clone different *Mus musculus* MPND fragments into the modified pET-28a vector that encodes an N-terminally His6-Tag, or the pGEX-4T-2 vector that encodes an N-terminally GST-Tag. According to prior publications [22,23], the tobacco etch virus (TEV) protease cleavage site can be used to remove the tags from these fusion proteins. By using site-directed mutagenesis and DNA sequencing, all MPND mutants were created and transformed into *Escherichia coli* BL21 (DE3) cells in order to express the proteins. At 37 °C, cells were cultured in the Luria–Bertani medium until the optical density at 600 nm reached 0.8–1.0. At this point, isopropyl β-D-thiogalactopyranoside (IPTG) was added to a final concentration of 0.3 mM. Further, induction was carried out at 18 °C overnight. The cells were then harvested by centrifuging at 4000× *g* for 10 min. Next, they were resuspended in lysis buffer (20 mM Tris-HCl pH 8.0, 1 M NaCl, 2 mM β-mercaptoethanol, and 5% glycerol) with 0.1% (*v*/*v*) Triton X-100 and 1 mM Phenylmethanesulfonyl fluoride (PMSF), and then disrupted by sonication. The cell lysates were clarified by centrifugation at 18,300 g min^−1^ for 40 min. The supernatant was filtered with a 0.45 μm filter membrane in order to remove cell debris before being loaded onto the His affinity column or GST affinity column (GE Healthcare, Uppsala, Sweden). The TEV protease was added to the eluate at a 1:10 (*w/w*, protease/protein) ratio at 4°C overnight to remove the N-terminal His-Tag or GST-Tag. The protein was further purified by ion exchange (UNO^TM^ S/Q, Biorad, Hercules, CA, USA) and size exclusion chromatography (SEC) using Superdex^TM^ 200 Increase 10/300 GL (GE Healthcare, Uppsala, Sweden). In addition, peak fractions were collected and assessed by SDS-PAGE. Then, the protein was concentrated at 5–10 mg/mL before crystallization. In order to obtain the protein–DNA complex, we directly mixed the purified protein and double-stranded DNA (dsDNA) at a 3:1 molar ratio, then incubated it on ice for 2 h. The complex was performed via SEC again for further purification and concentrated to about 5 mg/mL for crystallization. All protein purification processes were carried out at 4 °C.

### 4.2. Protein Crystallization and Data Collection

The crystallization trials of the apo–MPND and the MPND–DNA complex were initially performed using the sitting-drop vapor-diffusion method, by mixing 1 μL protein solution with 1 μL reservoir solution with various commercial crystallization kits in 48-well plates. The DNA oligonucleotides (top strand: 5′–CAGCAACAGAAGAGGATCT–3′, bottom strand: 5′–GAGATCCTCTTCTGTTGCT–3′) used for crystallization were dissolved in the buffer containing 10 mM HEPES pH 7.2. Annealing was then performed by heating the mixture of the two oligonucleotides at 95 °C for 10 min and slowly cooling to room temperature for 3 h. After a long period of optimization, the high-quality apo–MPND crystals were finally obtained in the reservoir buffer containing 25% PEG 4000, 0.1 M Bis-Tris pH 6.5, 0.2 M ammonium acetate at 4 °C. Further, the condition of the obtained protein–DNA complex crystals was recorded at 25% PEG 4000, 0.1 M sodium citrate pH 5.6, 0.2 M ammonium acetate at 16 °C. For the purposes of data collection, all crystals were cryoprotected in mother liquor supplemented with 25% (*v*/*v*) glycerol and flash-frozen in liquid nitrogen before data collection. The X-ray diffraction data were collected on beamlines BL10U2 and BL18U at the Shanghai Synchrotron Radiation Facility (SSRF). Moreover, the data were indexed, integrated, and scaled using HKL2000 [24]. Lastly, the data collection and processing statistics are summarized in Table 1.

### 4.3. Structure Determination

The atomic coordinates of CcrM (PDB ID: 6PBD) were used as a model to solve the apo–MPND structure by molecular replacement methods with the Molrep program in the CCP4 suite [16,19], based on extensive efforts of deleting different chains. The model was then built using the COOT [25] program and refined using REFMAC5 [26,27] in iterative cycles. The collection of the MPND–DNA complex crystals was initially satisfactorily indexed and integrated into the monoclinic space group *P*2_1_. However, the analysis demonstrated that this was a pseudo-space group brought about by the presence of DNA, which then turned to the lower symmetry triclinic space group *P*1. Thus, the apo–MPND structure was used as a search template with the molecular replacement method in order to determine the MPND–DNA complex structure in the *P*1 crystal form. The original density map clearly showed the shapes of the nucleic acids. Then, we matched the electron density map with the standard Β-type nucleic acid. We used the COOT program to build the model and then refined it using REFMAC5. PyMOL was used to create all structural figures in this article [28].

### 4.4. Electrophoretic Mobility Shift Assays

The EMSA experiments were performed to detect the binding ability of MPND or its mutants with single-stranded or double-stranded DNAs. As previously mentioned, annealing was used to create double-stranded DNAs. In addition, the buffer (20 mM HEPES pH 7.2, 100 mM NaCl, 2 mM Dithiothreitol (DTT), and 0.1% (*v*/*v*) NP-40) was added to a reaction mixture comprising 20 μM protein and 5 μM FAM-labeled dsDNA or ssDNA. Then, the reaction mixture was incubated for 30 min on ice. Following incubation, each sample was added to 5% (*v*/*v*) glycerol, then separated on a 6% native polyacrylamide gel in 0.5 L of TBE buffer (45 mM Tris-HCl pH 8.0, 45 mM boric acid, and 1 mM ethylenediaminetetraacetic acid) at 160 V for about 30 min. The gel was scanned with the ChemiDoc MP Imaging System (Biorad, Hercules, CA, USA) at 520 nm. Moreover, the oligonucleotides used in this study are shown in Table S1.

### 4.5. Small-Angle X-ray Scattering

Small-angle X-ray scattering (SAXS) measurements were performed on the beamline BL19U2 at the SSRF, following previously published methods [22,23]. All proteins were subjected to size exclusion chromatography in a buffer containing 20 mM HEPES pH 7.2, 100 mM NaCl, and 2 mM β-mercaptoethanol. We used a series of protein concentrations (60 μL) for the SAXS and collected the data at 1.03 Å with a distance of 2.68 m from the detector. Further, BioXTAS-RAW software (version 1.6.0) was used to process individual data [29]. In addition, FoXS was used to compare the scattering models of the MPND and MPND–DNA complex with experimental data [30]. Lastly, the data collection statistics are summarized in Supplementary Table S2 [31].

### 4.6. Microscale Thermophoresis (MST)

MST was used to calculate the binding affinity between the MPND and DNA. The oligonucleotides were labeled with 6-carboxyfluorescein (FAM) and annealed similarly to the oligos that were used for crystallization. Following the manufacturer’s instructions, both the wild-type and mutated MPND were mixed with DNA in a buffer containing 20 mM HEPES pH 7.2 and 100 mM NaCl, 0.1% (*v*/*v*) NP-40. Next, we loaded the samples into silica capillaries after 5 min of incubation at room temperature, and then measured temperature-induced fluorescence changes on the Monolith™ (NanoTemper) at 22 °C while using 20% LED and 40% MST power. The data analyses were performed using the NTA analysis software (NanoTemper Technologies).

### 4.7. Pull-Down Assay

The MPND constructs fused with the MBP-tag were purified using the appropriate affinity columns. The concentrations of MBP-MPND, histones, and nucleosomes used in the experiment were 0.5 μM, 2.1 μM, and 1.5 μM, respectively. We incubated MBP–MPND with histones or nucleosomes for 3 h at 4 °C in buffer containing 20 mM HEPES (pH 7.2), 0.15 M NaCl, and 1 mM DTT in the presence of amylose resin agarose beads. The resin was extensively rinsed with the same buffer to remove unbound or nonspecifically bound proteins. Proteins left on the beads were separated by SDS-PAGE, followed by Coomassie Bright Blue staining.

## Figures and Tables

**Table 1:** Data collection and refinement statistics of apo–MPND and MPND–DNA complexes.

	Apo–MPND	MPND–DNA Complex
	(PDB ID: 7YDT)	(PDB ID: 7YDW)
Data collection		
Space group	*P*2_1_2_1_2_1_	*P*1
Cell dimensions		
a,b,c (Å)	37.68, 62.84, 112.6	37.78, 62.92, 69.09
α,β,γ (˚)	90, 90, 90	72.58, 89.88, 89.98
Resolution (Å) ^a^	50.0–2.06	50.0–2.47
	(2.10–2.06)	(2.51–2.47)
Rmerge	11.5% (49.6%)	10.5% (32.5%)
*I*/σ	13.3 (2.0)	9.5 (2.0)
Completeness (%)	97.6 (83.7)	96.0 (80.8)
Total no. of reflections	97,574	71,276
Unique reflections	17,365	21,839
Redundancy	5.8 (5.1)	3.4 (3.1)
CC_1/2_	0.950 (0.883)	0.968 (0.894)
Refinement		
Resolution (Å)	50.0–2.06 (2.11–2.06)	50.0–2.47 (2.53–2.47)
No. of reflections	16,061	19,835
*R*_work_/*R*_free_ (%)	23.0/24.9	24.5/25.4
No. of atoms		
Protein	1427	2841
Ligand/ions	0	409
Water	62	161
Average *B*-factors (Å^2^)		
Protein	37.94	50.42
Ligand/ion	0.00	63.17
Water	39.13	52.33
rms deviations ^b^		
Bond lengths (Å)	0.002	0.003
Bond angles, °	1.149	1.181
Ramachandran plot, % ^c^	94.9/5.1/0	94.1/5.9/0

^a^ Statistics for highest resolution shell. ^b^ Root-mean-square deviations. ^c^ Residues in favored, allowed, and outlier regions of the Ramachandran plot, respectively.

## Data Availability

For apo–MPND and the MPND–DNA complex, the final coordinates and structure factors have been deposited in the Protein Data Bank (PDB) under accession codes 7YDT and 7YDW, respectively.

## Supplementary Materials

The following are available online at https://www.mdpi.com/article/10.3390/ijms24043368/s1. References [17,29,30,32,33] are cited in supplementary materials.
