# Supplementary material for: Structures of MPND Reveal the Molecular Recognition of Nucleosomes
Source: Int J Mol Sci. 2023 Feb 8;24(4):3368. doi: 10.3390/ijms24043368 (PMC9963953; doi:10.3390/ijms24043368)
Supplement: Supplementary file 1 [file ijms-24-03368-s001.zip › ijms-2148774-supplementary.pdf]

Supplementary Information

Structures of MPND Reveal the Molecular Recognition of Nucleosomes

Meiting Yang, Xiaorong Li, Zizi Tian, Lulu Ma, Jun Ma, Yunlong Liu, Guohui Shang, Ailing Liang, Wei Wu and Zhongzhou Chen

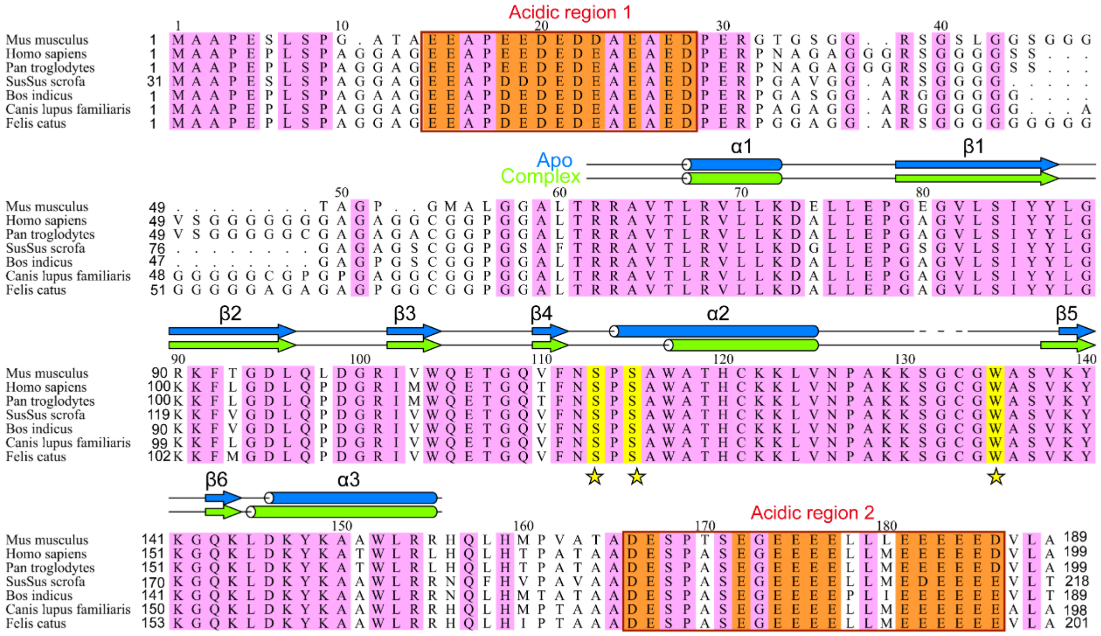

Figure S1. Structure-based sequence alignment of MPND proteins from different species. Secondary structural elements of the DNA-free and DNA-bound MPND structures were calculated using DSSP and colored in blue and green, respectively. Cylinders and arrows represent α-helices and β-strands, respectively. The invariant residues between different species are shown in pink. Acidic residues in the two acidic regions are shown in orange. Yellow stars indicate residues used in mutational analyses. The sequences used are: MPND from Mus musculus (NP_080806.4); Homo sapiens (NP_116257.2); Pan troglodytes (XP_016790200.1); Sus scrofa (XP_013850219.2); Bos indicus (XP_019819993.1); Canis lupus familiaris (XP_013977660.2); Felis catus (XP_023099774.2). The ALINE program [1] was used to prepare the figure. Note that only chain D in the complex has conformational change of α2.

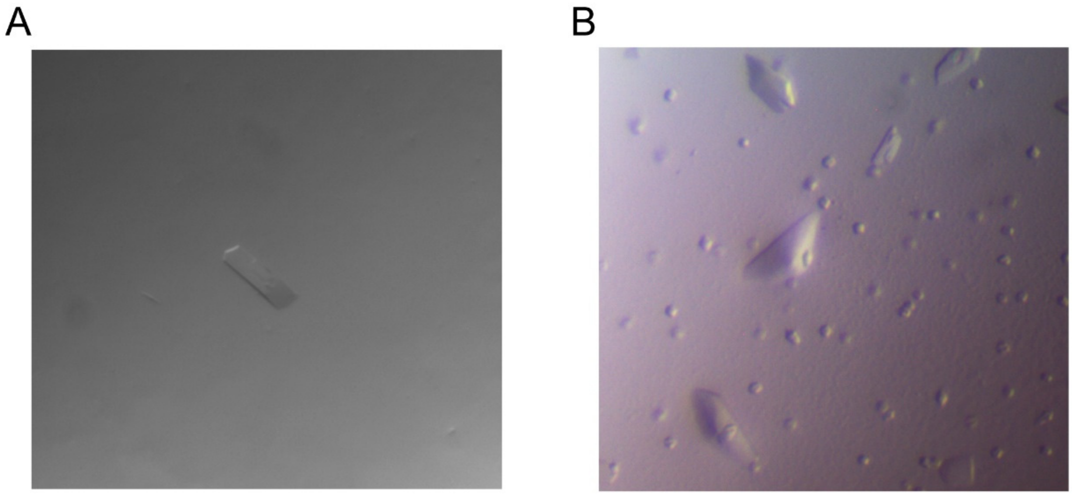

Figure S2. Crystals for diffraction experiments. Crystals of apo-MPND (A) and MPND-DNA complex (B).

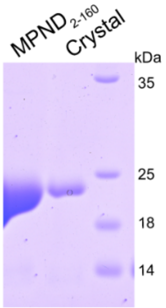

Figure S3. The apo-MPND proteins before and after crystallization. The sizes of these two proteins were equal (~17 kDa), which meant that the missing residues in the apo-MPND structure were due to their flexibility.

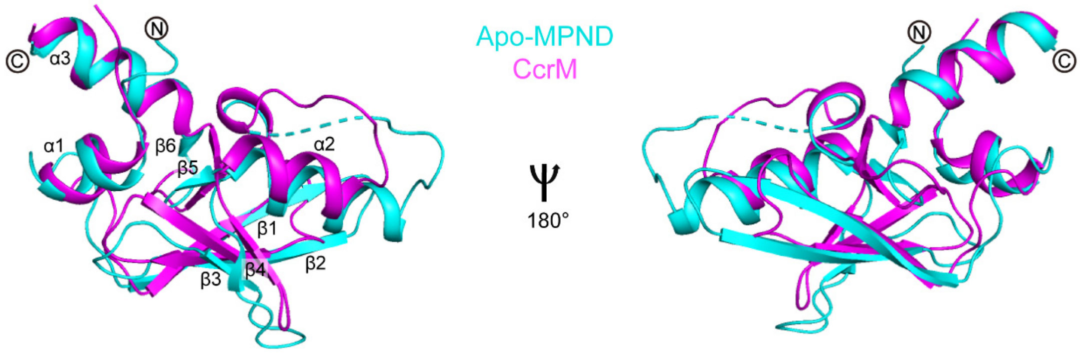

Figure S4. Structural superimposition of apo-MPND (cyan) onto DNA methyltransferase protein (magenta) (CcrM, PDB ID: 6PBD, Z = 10.4, RMSD = 1.9 Å) [2].

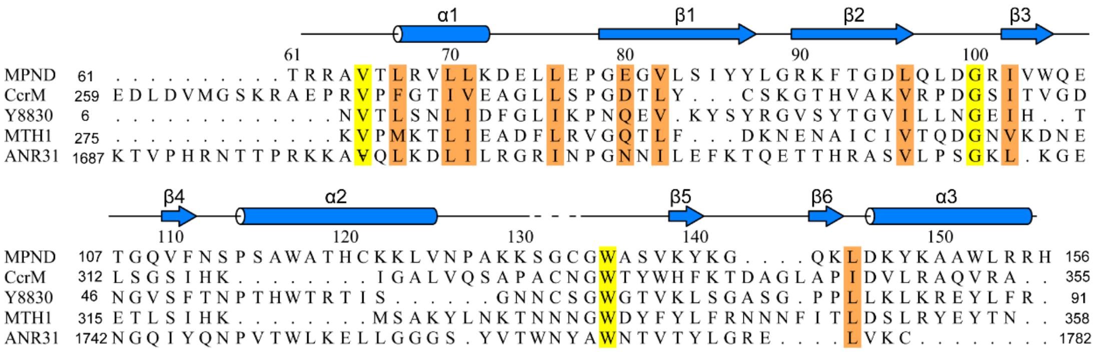

Figure S5. Structure-based sequence alignment of five different proteins containing RAMA domain. The sequences used in this figure: MPND (NP_080806.4); CcrM (WP_010918266.1); Y8830 (XP_645093.1); MTH1 (WP_021035599.1); ANR31 (NP_001357857.1). The ALINE program [1] was used to prepare the figure.

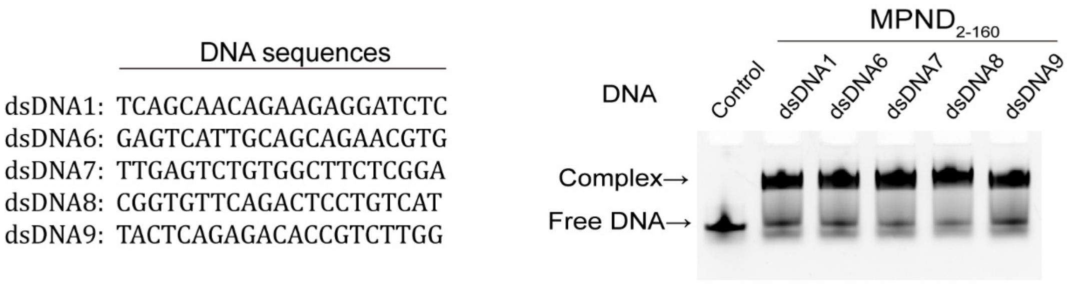

Figure S6. EMSA of MPND with different DNA sequences. All DNA sequences did not have obvious conserved motifs or other features such as GC or AT enrichment. Note that only the sequences of the up-strand of dsDNA are shown.

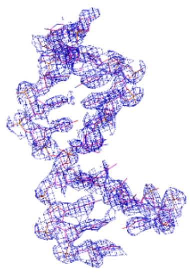

Figure S7. The composite simulated-annealing sigma-A-weighted 2mFo − DFc electron density map of DNA in the MPND-DNA complex was shown at 1.0 σ.

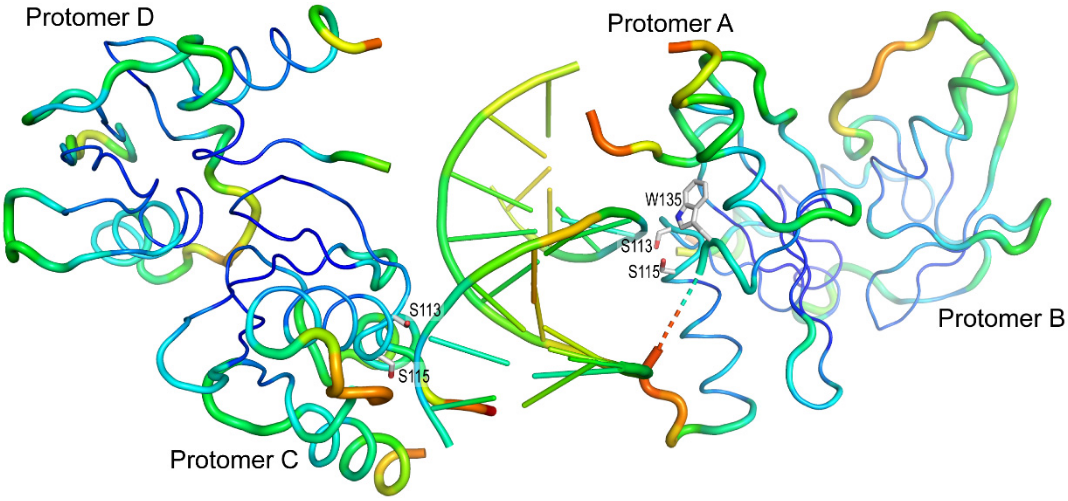

Figure S8. Temperature factor of the MPND-DNA complex, and color-coded based on the calculated B-factor. The colors ranged from blue to red, corresponding to increasing fluctuations. The key residues were displayed in the stick form.

Table S1. Oligonucleotides used in this study.

ID	Sequence (5'-3')	
ssDNA1-F	TCAGCAACAGAAGAGGATCTC	
ssDNA1-R	GAGATCCTCTTCTGTTGCTGA	
ssDNA2-F	GATGCAAGCATCAGCAACAGAAGAGGATCTCAGGTGCAGCGC	
ssDNA2-R	GCGCTGCACCTGAGATCCTCTTCTGTTGCTGATGCTTGCATC	
ssDNA3-F	AGCAACAGAAGAGGATCT	
ssDNA3-R	AGATCCTCTTCTGTTGCT	
ssDNA4-F(6mA)[1]	AGCAAC**A**GAAG**A**GGATCT	
ssDNA4-R	AGATCCTCTTCTGTTGCT	
ssDNA5-F	GCAACAGAAGAGGAT	
ssDNA5-R	ATCCTCTTCTGTTGC	
Bubble3-F	TCAGCAACAGAAGAGGATCTC	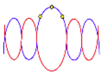
Bubble3-R	GAGATCCTCTGAGGTTGCTGA	
Bubble6-F	TCAGCAACAGAAGAGGATCTC	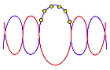
Bubble6-R	GAGATCCGAGGAGGTTGCTGA	
Bubble9-F	TCAGCAACAGAAGAGGATCTC	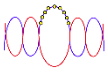
Bubble9-R	GAGATCCGAGGAGTGGGCTGA	
Bulge3-F	TCAGCAACAGAAGAGGATCTC	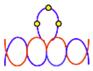
Bulge3-R	GAGATCCTCTGTTGCTGA	
Bulge6-F	TCAGCAACAGAAGAGGATCTC	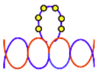
Bulge6-R	GAGATCCGTTGCTGA	

[1]6mA: Adenine is methylated at the 6th position of the purine ring (N6) in DNA, represented by **A**.

Table S2. SAXS data-collection and analysis.

Parameter	**Value**
Data collection	
Instrument	SSRF BL19U2
Wavelength (Å)	1.03
Beam size (μm)	320 × 43
Exposure time (s)	1.0
Camera length (m)	2.68
Temperature (K)	283
Concentration (mg/ml)	1.0-5.0
Software employed	
Primary data reduction	BioXTAS-RAW [3]
Validation	FoXS [4]
Three-dimensional graphics	PyMOL [5]

Supplementary references:

1. Bond, C.S.A.W. Schüttelkopf. ALINE: a WYSIWYG protein-sequence alignment editor for publication-quality alignments. *Acta Crystallogr. D* **2009**, *65*, 510-512.
2. Horton, J.R.; C.B. Woodcock.; S.B. Opot.; N.O. Reich.; X. ZhangX. Cheng. The cell cycle-regulated DNA adenine methyltransferase CcrM opens a bubble at its DNA recognition site. *Nat. Commun.* **2019**, *10*, 4600.
3. Nielsen, S.S.; K.N. Toft.; D. Snakenborg.; M.G. Jeppesen.; J.K. Jacobsen.; B. Vestergaard.; J.P. KutterL. Arleth. BioXTAS RAW, a software program for high-throughput automated small-angle X-ray scattering data reduction and preliminary analysis. *J Appl Crystallogr* **2009**, *42*, 959-964.
4. Schneidman-Duhovny, D.; M. Hammel.; J.A. TainerA. Sali. Accurate SAXS profile computation and its assessment by contrast variation experiments. *Biophys. J.* **2013**, *105*, 962-974.
5. Mooers, B.H.M. Shortcuts for faster image creation in PyMOL. *Protein Sci.* **2020**, *29*, 268-276.
